# Microbial innovations for sustainable water treatment

**DOI:** 10.1093/sumbio/qvag004

**Published:** 2026-03-09

**Authors:** Francis Hassard, Thomas Thompson, Victor Castro-Gutierrez

**Affiliations:** Cranfield Water Science Institute, Cranfield University, College Way, Bedford, MK43 0AL, United Kingdom; School of Pharmacy, Queen’s University Belfast, Medical Biology Centre, 97 Lisburn Road, Belfast BT9 7BL, United Kingdom; Environmental Pollution Research Center (CICA), University of Costa Rica, Montes de Oca 11501, Costa Rica

Access to clean and safe water is increasingly challenged by the converging forces of population growth, rapid urbaniaation, aged infrastructure, climate-driven hydrological extremes, and escalating anthropogenic pressures on finite freshwater resources. While the UN Sustainable Development Goal (SDG) 6 is often colloquially summarized as “clean water and sanitation,” its official scope is systemic, encompassing safe drinking water, effective sanitation, improved ambient water quality, and the protection of water-related ecosystems (United Nations Department of Economic and Social Affairs 2026). Microorganisms are the invisible engines central to these objectives, while also representing potential threats that need to be suitably managed. Microbial metabolic diversity underpins nutrient cycling, contaminant transformation, and the resilience of both engineered and natural water systems.

This themed collection in Sustainable Microbiology brings together microbial innovations that deliver effective water treatment solutions while reducing environmental footprints. Across the water cycle, the collection highlights approaches that minimize chemical inputs and energy demand, improve system robustness, and support the paradigm transition from wastewater treatment to water resource recovery (van Loosdrecht and Brdjanovic 2014).

## Microbial process innovations: efficiency and disinfection

A cornerstone of this transition is the move from traditional chemical-intensive disinfection approaches to more targeted advanced technologies capable of tackling recalcitrant micropollutants and unwanted biofilms (e.g. in distribution systems or on membranes) that can shelter pathogens and contribute to fouling, which are distinct from biofilms intentionally harnessed for treatment in many wastewater bioprocesses. Cold atmospheric plasma (CAP) is an emerging, nonthermal technology that generates reactive oxygen and nitrogen species (RONS) at the gas-liquid interface; these species can inactivate pathogens, weaken biofilm matrices, and contribute to the degradation of complex pollutants (e.g. pharmaceuticals, dyes, antibiotics), while reducing reliance on externally supplied chemical reagents. At the molecular level, RONS can oxidize cellular components and extracellular polymeric substances and transform organic contaminants to a range of intermediates; identifying transformation products and ensuring no harmful by-products remains an important research need. In this collection, Maybin et al. (2024) review CAP for enhanced water purification, summarizing reported treatment performance and highlighting key barriers for implementation, including scale-up and economic feasibility.

## Wastewater as a sentinel: advanced monitoring

Sustainable water management depends on both treatment performance and timely detection of risk. Wastewater-based epidemiology has matured into a powerful noninvasive tool for public health, as demonstrated during the SARS-CoV-2 pandemic (Hassard et al. 2023). Beyond viral pathogens, surveillance is critical for tracking the “silent pandemic” of antimicrobial resistance (AMR). Ochman et al. (2024) survey tetracycline resistance genes across primary and secondary stages of a wastewater treatment plant, detecting 17 of 22 targeted genes. Using epicPCR to link genes to hosts, the study shows tetracycline-resistance genes are less host-restricted than assumed, including novel host-gene associations consistent with ARG spread across diverse taxa. Alfahl et al. (2024) undertook a PRISMA-guided scoping review of AMR detection methods in water environments (115 studies), mapping where AMR is monitored and which methods dominate. It reports increasing use of PCR and metagenomics for sensitive, comprehensive detection. Other studies in Sustainable Microbiology show promise for culture-independent surveillance beyond wastewater: Claveau et al. (2024) used flow cytometry fingerprinting (rapid single-cell measurement of light scatter and fluorescence that generates a community fingerprint) as a source-to-tap tool for microbial event detection across drinking-water treatment and service reservoirs. The authors show that microbial fingerprint deviations can indicate operational disturbances and potentially poorer microbial quality.

## Ecological design and resource recovery

Sustainability in water treatment is increasingly framed as an ecological design challenge: harnessing nature’s own capacity for adaptation and evolution. This includes nature-based solutions (NBS) that utilize specific microbial traits to bioremediate contaminated environments, reducing the need for physicochemical interventions. Ahmed et al. (2026) synthesized how boron-tolerant bacteria (including Bacillus, Lysinibacillus and Microbacterium) can support sustainable bioremediation by reducing boron bioavailability and improving plant tolerance in phyto-bioremediation systems. It outlines key tolerance mechanisms (e.g. efflux, bioaccumulation, quorum sensing and redox regulation; transporter families) and highlights knowledge gaps and future prospects for deployment. This themed collection also features articles on bacteria within wastewater treatment systems optimized for micropollutant degradation. Looking ahead, urgent priorities include climate-resilient and low-emission treatment, management of persistent contaminants and transformation products, and scalable surveillance for pathogens and AMR.

The intersection of microbiology and environmental biotechnology offers practical routes to address escalating water demands while protecting ecosystems and public health. By bringing together work on microbial process innovations, NBS, resource recovery, and surveillance, this collection aims to support the advances needed to achieve SDG 6. We thank all contributors and reviewers for shaping the collection and hope it stimulates further interdisciplinary collaboration across research, practice, and policy.

## Data Availability

No datasets or software code was generated for this editorial.
